# The Teeth

**Published:** 1875-01

**Authors:** 


THE TEETH.—We don't like to allow an issue of our magazine to go to the world unless it contains a few words of counsel and warning upon the necessity of taking care of the teeth. Not a day passes in which we are not forcibly reminded of the terrible thoughtlessness of the human family in this important particular. More than half the people whom we meet have a bad-smelling mouth, and teeth the beauty of which has been seriously injured by want of care. It is no use to say "Why, teeth decay naturally," for teeth never decay naturally. Decay in teeth is simply solution, and nature does not make teeth for the simple purpose of dissolving them in the juices of the mouth. The talk about parasites and destructive microscopical insects who feed on the teeth, and are never so happy as when lunching on a molar, is all very well, but is in the main fallacious; for there are no animals about the teeth which require a weapon more formidable for their destruction than a good firm tooth-brush, dipped in water, and charged with a little soap or chalk. If parents will provide their children with hard, flexiible brushes, and see that they are used invariably after eating, they will do them a more valuable service than by leaving them many thousand dollars in money.

---

As the world gets older and the people more numerous, the striving for bread becomes more desperate; and in large cities it is already a struggle for life. "I must get bread or die," has fallen from many a quivering lip. This accounts for no little of the crime in the land. Good men should consider this, and, in bringing criminals to punishment, should seek to know whether the crime is impelled by a bad heart or by dire want.

TWO EXCELLENT HOTELS.—Boston, Mass., has always been a comfortable place to live in, not only for the housekeeper but for the comparatively transient person who is compelled to find "Life's warmest comforts at an inn." Perhaps its reputation has been acquired by no influence so potent as the quality of its hotels. Upon the exact site now occupied by one of these famous establishments the present writer was born, and it will not appear strange that he should take an interest in all that belongs to it. The TREMONT HOUSE and the REVERE HOUSE are strictly first-class houses, having every comfort and convenience, and being conducted in such a manner as to make all guests feel that their wants and needs are the chief thought of the proprietors and attendants. We are impelled to speak thus warmly of these hotels because of our actual knowledge of their merits.

---

HOUSEKEEPERS should recollect that it is the penny saved, more than the penny earned, that enriches; it is the sheet, or blanket, or garment darned when the first thread breaks that wears the longest; it is the dampers closed when the cooking is done that stops some unnecessary dollars from dropping into the coal bin; it is the lamp or gas turned low when not in use that gives you pin money for the month; it is taking care to see that the coffee is browned instead of being burned or merely dried, that it is ground fine instead of being coarsely cracked, which makes three spoonfuls go as far as a cupful; that, in short, it is eternal vigilance in little things which makes the difference between the thoughtless poor and the forehanded middle-class.

---

If you would be well, lead an active life in the open air.

